# Organizational factors affecting length of stay in the emergency department: initial observational study

**DOI:** 10.1186/s13584-015-0035-6

**Published:** 2015-10-15

**Authors:** Osnat Bashkin, Sigalit Caspi, Rachel Haligoa, Sari Mizrahi, Ruth Stalnikowicz

**Affiliations:** Department of Public Health, School of Health Sciences, Ashkelon Academic College, Ashkelon, Israel; Department of Industrial Engineering & Management, Azrieli College of Engineering, Jerusalem, Israel; Department of Emergency Medicine, Hadassah Medical Center–Mt. Scopus, Jerusalem, Israel

**Keywords:** Emergency department, Length of stay, Organizational factors, Ishikawa causal diagram

## Abstract

**Background:**

Length of stay (LOS) is considered a key measure of emergency department throughput, and from the perspective of the patient, it is perceived as a measure of healthcare service quality. Prolonged LOS can be caused by various internal and external factors. This study examined LOS in the emergency department and explored the main factors that influence LOS and cause delay in patient care.

**Methods:**

Observations of 105 patients were performed over a 3-month period at the emergency room of a community urban hospital. Observers monitored patients from the moment of entrance to the department until discharge or admission to another hospital ward.

**Results:**

Analysis revealed a general average total emergency department LOS of 438 min. Significant differences in average LOS were found between admitted patients (Mean = 544 min, SD = 323 min) and discharged patients (Mean = 291 min, SD = 286 min). In addition, nurse and physician change of shifts and admissions to hospital wards were found to be significant factors associated with LOS. Using an Ishikawa causal diagram, we explored various latent organizational factors that may prolong this time.

**Conclusions:**

The study identified several factors that are associated with high average emergency department LOS. High LOS may lead to increases in expenditures and may have implications for patient safety, whereas certain organizational changes, communication improvement, and time management may have a positive effect on it. Interdisciplinary methods can be used to explore factors causing prolonged emergency department LOS and contribute to a better understanding of them.

## Background

Emergency medicine is the medical specialty that links primary care and specialist care treating unexpected illness and injury. Therefore, it must be available 24 h a day as an essential component of a healthcare system [1]. In this work environment, emergency department (ED) staff face unique challenges such as treatment of patients arriving to the department with dynamic and unexpected states of illness, dealing with uncertainty regarding patient medical histories, and the need for time-dependent and triage-based decision making [1], all of which are often accompanied by high financial costs to healthcare systems and a pressure to be economically efficient [2]. According to the Institute of Medicine, one of the most common weak points of EDs is crowding, and it is important to understand the causes, effects, and prevention strategies for this [3]. Overcrowding diminishes the capability of the EDs to manage and provide immediate access and stabilization for patients who have an emergency medical condition [4]. In a study analyzing crowding [5], researchers found three main factors contributing to it: *input factors* reflected sources and aspects of patient inflow, *throughput factors* reflected bottlenecks within the ED, and *output factors* reflected bottlenecks in other parts of the healthcare system that can affect the ED.

Patient length of stay (LOS) is a key measure of ED throughput and has been identified as a major cause of bottlenecks and overcrowding [6]. Previous studies [7, 8] have also shown that, among other factors, extended lengths of stay increase the likelihood of patients leaving the ED without being seen by a physician. In a study that examined ED LOS [9], researchers associated it with excess inpatient LOS for patients admitted from the ED. Excess inpatient LOS was defined as: exceeding the stated benchmark for the relevant diagnosis-related group. They found that compared with patients who stay in the ED for 4–8 h, those who remain in the ED for 8–12 h are 20 % more likely to stay in hospital longer than the national average for the relevant admission problem. Moreover, this number rose to 50 % if emergency department LOS was greater than 12 h.

Another study [10] examined one of the direct factors influencing emergency department LOS known as “access block,” which refers to the situation in which patients requiring an emergency hospital admission remain more than 8 h in the emergency department due to a lack of access to appropriate hospital inpatient beds [9]. The study [10] examined the relationship between access block in the ED, defined as the total time exceeding eight hours from a patient’s initial arrival in the ED to transfer to another department and inpatient LOS. Results revealed that 7.7 % of 11,906 patients experienced access block. In addition, the mean LOS was 4.9 days in those who experienced access block compared with 4.1 days in those who did not. Subgroup analysis showed that the effect of access block varied across severities of illness and diagnoses. For example, the mean LOS was 3.9 days in patients with cardiac diagnosis who experienced access block compared with 5.6 days in cardiac patients who did not experienced access block.

In another study [6] researchers identified several independent variables that have been associated with ED LOS. Researchers found that triage level, diagnostic tests, and consultations have a major effect on it. Waiting time to see a physician was also found to be one of the variables influencing this time. Patients in intermediate triage levels III and IV (non-urgent patients) spent the longest waiting times for nurse and physician assessment and the longest ED lengths of stay. In addition, the study revealed that the use of diagnostic imaging and laboratory tests were associated with longer LOS, varying with the specific tests ordered. Specialty consultation was also associated with prolonged LOS, and this effect was highly variable depending on the service consulted.

Our study objectives were to examine organizational factors affecting ED LOS. Organizational factors are structural, cultural and policy related characteristics of the organization [11]. The negative consequences of organizational processes (that is, decisions concerned with planning, designing, policy making, regulating) can create a local conditions that promote human errors (for example understaffing, high workload) [12]. In the study, we also used an Ishikawa fishbone diagram, a quality management tool, to explore these factors and to offer an organizational perspective of their effect on prolonged ED LOS. The Ishikawa fishbone diagram is an analysis tool that provides a systematic view of the effects and the causes that create or contribute to those effects. The diagram is considered one of the seven basic tools of quality control and is commonly used in manufacturing industry, as well as in healthcare settings. Using this tool, all actions, events, and environmental circumstances that may explain why the problem may have occurred are identified. The head of the diagram represents the main problem and the potential causes of the problem, usually derived from brainstorming sessions or research, are indicated in the ‘fish bones’ of the diagram [13].

## Methods

### Study field

Observations were made in the emergency department of a community hospital in Israel. The emergency department include three branches: internal, pediatrics and surgical/orthopedic. The observations took place at the internal and the surgical/orthopedic branches. The department admits approximately 60,000 patients per year. ED medical staff work in three shifts: a morning shift from 07:00 to 15:00, an evening shift from 15:00 to 23:00, and a night shift from 23:00 to 07:00. There are 7 registered nurses and 4 physicians scheduled for each shift. The department has 38 patient beds.

### Data collection and tools

Two senior Industrial Engineering & Management students performed observations in the ED after receiving consent from medical staff members. The observers performed their observations separately. They observed the patient during his/her stay in the ED. Prior to data collection, the observers met several times with the ED staff and among themselves to develop rapport and consistency in the observation process. The observers monitored randomly 105 patients who arrived in the ED during a 9-day period, in the morning and evening shifts. Total LOS was recorded for each of the patients, from the moment of entering the ED until discharge or admission using a wristwatch.

Data collected included: time of registration, time of nursing and time of physician assessment, time of medical decision making (discharge vs. admittance), and use of specialty consultation and ancillary services, as well as time of departure. Admitted patients were not considered to have departed from the ED until they were physically transported out of the ED to the hospital inpatient ward or another patient care facility. Observers recorded in addition to time, any relevant aspect of the process of care during the patients stay in the ED that may have had effect on ED LOS.

### Data analysis

SPSS statistical software was employed for statistical analyses and for assessing quantitative trends. Variable relationships were described using descriptive statistics. Mean times (minutes) measured were compared using unpaired two sample t-tests.

## Results

**Fig. 1:**
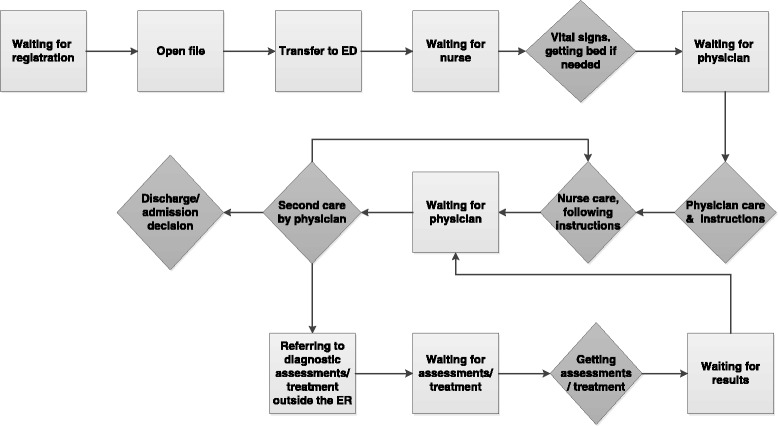
Workflow of patient care in the ED

In the first stage of data analysis, we mapped the workflow of the emergency department process from registration to discharge or hospital admission in order to examine the different steps involved in patient care. The workflow diagram is presented in Fig. 1.

The light grey squares show waiting times that affected the overall ED LOS. The dark grey diamonds demark steps that are essential to patient and to process flow. As can be seen in Fig. 1, some steps are repeated if a patient is required to undergo diagnostic tests, as well as to be examined several times by physicians. Assessments or treatments outside the ER infrastructure can involve processes that cause more waiting times and may lengthen the total ED LOS. Nevertheless, the diagram presented in this figure illustrates that waiting times occur at various steps in the process of the ED stay extending LOS; therefore, we decided to examine the source factors influencing ED LOS.

**Fig. 2:**
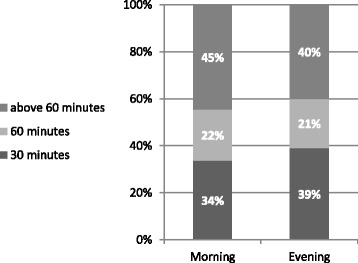
Time from registration to first physician examination

As mentioned above, observers monitored 105 patients who arrived in the ED during a 9-day period: 52 % were registered during the morning shift and 48 % during the evening shift. Figure 2 presents the waiting times of patients from registration in the ED until the first physician examination.

Figure 2 shows that among patients registered in the ED during the morning shift (07:00 to 15:00), 45 % waited over an hour before their first physician examination. Among patients registered in the ED during the evening shift (15:00 to 23:00), 40 % waited over an hour before their first physician examination.

Analysis of data regarding medical staff shift changes revealed that 64 % of the patients been observed experienced a shift change of nurses during their stay. The average total LOS for these patients was 564 min (SD = 339 min) compared to an average total LOS for patients who did not experienced a shift change of nurses, of 185 min (SD = 83 min). Unpaired two sample t-tests revealed significant differences in total LOS between patients experiencing nurse shift changes and patients who did not [t(103) = 6.68, ρ < .01]. Similar results were found with regard to physician shift changes: 61 % of patients observed experienced a shift change of physicians. Our analysis revealed that the average total LOS for these patients was 563 min (SD = 350 min) compared to an average total LOS for patients who did not experience this change of 257 min (SD = 223 min). Unpaired two sample t-tests revealed significant differences in total LOS between patients experiencing a shift change of physicians and those who did not [t(103) = 5, ρ < .01].

**Fig. 3:**
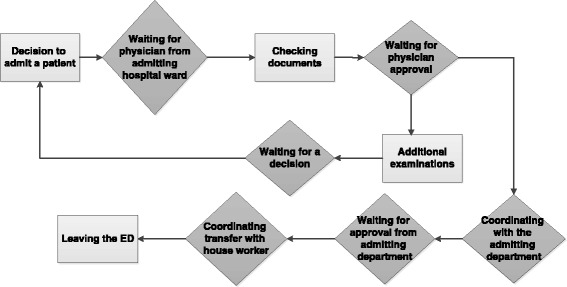
Workflow of patient admission in the ED

In this study, 44 % of the patients under observation were admitted to the hospital, while 56 % were discharged. Once the decision to admit a patient was made, the median time necessary to physically transfer the patient out of the ED and to the appropriate hospital ward was 514 min. The median time from a decision to discharge a patient until the patient left the ED was 203 min. The average total LOS for admitted patients was 544 min (SD = 323 min) and for discharged patients 291 min (SD = 286 min). Unpaired two sample t-tests revealed significant differences in total LOS (from registration in the ED to admission/discharge) between admitted patients and discharged ones [t(103) = 4.19, ρ < .01]. These differences in total LOS between admitted patients and discharged patients led us to assume that the time of the decision to admit a patient may be the beginning of a bottleneck process because it involves interdepartmental arrangement. Examination of flows from the moment of decision to admit a patient clarified the problematic steps of this process. Figure 3 presents a workflow diagram of the admission process in the ED.

As can be seen in Fig. 3, the process of admission of the patient often creates a bottleneck related to the admitting hospital ward. The steps in the process that begin with the decision to admit the patient and end with obtaining approval from the physician of the admitting hospital ward are the most time consuming. The process presented in Fig. 3 represents 43 % (233 min.) of the total ED LOS of admitted patients.

Additional analyses were performed in order to examine if there are any differences between the admitting hospital wards in the total ED LOS. Analysis of variance (Anova) was performed in order to compare the mean ED LOS of the patients admitted to six hospital wards (gynecology, orthopedic, chirurgical medicine, internal medicine, and geriatric departments, and the cardiology unit). Analysis revealed significant differences between the average ED LOS of patients admitted to these departments [F (5) =2.7, ρ < .05]. The highest average ED LOS were for patients admitted to the orthopedic department (Mean = 382 min, SD = 318 min) and the internal medicine department (Mean = 259 min, SD = 170 min).

**Fig. 4:**
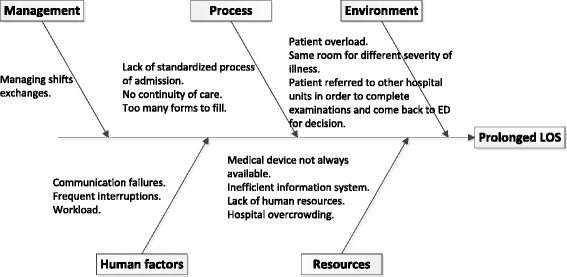
Ishikawa diagram identifying causes of prolonged ED LOS

We used an Ishikawa diagram [13] to identify the factors causing the overall effect of extended ED LOS. This diagram is shown in Fig. 4.

The factors presented in Fig. 4 were revealed during the observation period and were classified into five categories, according to the categories usually classified using the method of Ishikawa diagram [13]: managerial, process, environmental, human, and resource factors. Classification of the factors into categories were made through a meeting with all ED staff members along with the researchers and the observers, and using a brainstorming process. The Ishikawa causal diagram enabled the consideration of organizational-related factors such as standardization and workflow, communication failures and workload, all of which can influence ED LOS.

## Discussion and Conclusion

High LOS may lead to crucial expenditures and may have implications on patient safety. Using prospective observational methods for data collection and an Ishikawa fishbone diagram for data examination, our study identified several factors associated with ED LOS.

Nurse and physician handover in shift changes appear to be among the chief factors with significant influence on ED LOS. In a study that examined handover in change of shifts in ED [14], researchers noted that one of the main considerations that distinguishes ED from other health services is the handover involved in the change of shifts. The content and needs related to this handover vary from patient to patient, and the incoming physician needs to develop the coordination of care for the patients, all of whom may have different and unrelated medical case scenarios and, thus, very different needs. This is in contrast to what occurs with most cross-coverage handovers in other hospital wards, in which most of the patients have similar medical case scenarios and lower clinical status changes and acuity. The incoming physicians in other hospital wards are most often familiar with the patients’ needs [14].

Another study [15] emphasized the complexity of ED handover in which standardization was very low, patient flow was unpredictable and varied, and the likelihood of the incoming provider having to interact with the case being handed over was very high. This is in contrast to inpatient wards where the handover is often more similar than different. Problematic handover at change of shift may have critical consequences causing delays in medical diagnosis, prolonged hospital LOS, and higher probability of adverse events in the ED [16].

Previous studies have shown that 84 % of treatment delays are later judged to be due to miscommunication; of these, 62 % are continuum-of-care issues associated with shift changes [17]. In our study, problems in continuum-of-care were found to affect ED LOS in another form, mainly among the admitted patients group. The process—from deciding to admit a patent until the patient was actually transferred from the ED to an inpatient hospital ward—took 43 % (233 min.) of the total ED LOS of admitted patients. Analysis of the admitting process using Ishikawa diagram [see Fig. 4] revealed several possible explanations to the prolonged waiting times, one of them being deficient communication. For example, when a decision to admit a patient to another hospital ward was made, the ED physician called the receiving inpatient physician to approve the decision. When the physician arrived in the ED, he examined the patient and his/her medical records and in some cases decided to request more diagnostic tests or medical examinations before approving admission. This took additional time, and the patient often had to go to other hospital units to have the tests and then return to the ED. The loop of calling the physician from the admitting department began again and often with a different receiving inpatient physician responding, who lacked the knowledge regarding the patient’s health condition (due to miscommunication) and thus requested more diagnostic tests be done and so on.

The literature suggests that lack of continuum-of-care in the process of patient admissions is derived from deficient communication among medical staff [18], and in our study, we found that it is associated with, and may have led to, prolonged ED LOS. Continuity of patient care is based on the effective transfer of information between medical staff members. A study [19] that examined the handoff process between ED medical staff and Intensive Care unit (ICU) medical staff revealed that there was no structured and consistent approach to how handovers actually occurred, and nurses from both ED and ICU lacked clarity as to when the actual handover process began. Nurses from both settings recognized the importance of the information given and received during handover and deemed it to have an important role in influencing the quality and continuity of care.

In a study examining the perceptions of ED physicians and hospitalizing physicians regarding handoff communication of patients transferred from ED to inpatient settings [20], researchers found that physicians perceived handoff communication as characterized by ambiguity about patients’ conditions and treatment. They found that poor communication practices and conflicting communication expectations presented barriers that exacerbated physicians’ information ambiguity. They noted that ED physicians and receiving inpatient physicians had different expectations about handoffs and those expectations influenced their interactions in ways that could result in communication breakdowns. Hospitalizing physicians expect ED physicians to produce definitive diagnoses, and admissions are delayed until confirmatory test results are provided, whereas emergency physicians might believe that their professional opinions are being questioned.

At the organizational level, prolonged ED LOS is often associated with hospital occupancy rate. Previous studies have demonstrated the effect of hospital overcrowding on ED overcrowding [21–23]. One of these studies [23] found that daily ED LOS for admitted patients increased 18 min when there was an absolute increase in hospital occupancy of 10 %. The ED LOS appeared to increase extensively when hospital occupancy exceeded a threshold of 90 %. Another study [24] revealed that crowding was associated with substantial delays in ED LOS across the four ED sites it examined. Moreover, crowding prolonged the ED LOS of high-acuity patients. In addition, researchers noted output factors, such as the number of patients admitted and the inpatient medicine occupancy rate, that were associated with significant delays in ED care. These findings, which support those in our study, showed that ED LOS was highest among two of the receiving inpatient departments (orthopedic and internal medicine departments), both of which are characterized by very high occupancy rates.

There are several limitations of the study regards external validity and generalizability. First, we analyzed a small number of ED visits in a limited period. Second, we focused on a single ED in Israel, which possibly functions differently from other EDs in Israel and elsewhere. The hospital participated in our study is a community hospital with limited capacity of admission beds. As a result, the standard route of emergency admission in the hospital studied is for an ED physician to ask an in-hospital consultant to see the patient and consider admission. It is possible that there is an association between the limited capacity of admission beds and the amount of diagnostic tests required by the receiving inpatient physicians before deciding to admit the patient. However the limited capacity of admission beds, cannot explain the association between prolonged ED LOS and shift changes found in our study. Thus, the relevance of deficient communication and lack of continuum-of-care to ED LOS is probably not limited to community hospitals with insufficient bed supply and/or hospitals where the physicians from the admitting departments have a substantial say in the process and timing of the admission decision.

Concerning the findings that showed prolonged LOS for patients who experienced a shift change, it is possible that the long stay itself leads patients to span the shift change, rather than the opposite. We cannot determine a cause and effect relationship between shift change and prolonged LOS, but the prolonged LOS for patients who experienced a shift change shown in our study emphasize the complexity of handover in shift change. In addition, we lack the data regarding the clinical characteristics and acuity of health status of the observed patients that may explain the differences in ED LOS, which were attributed to organizational factors.

Our study results demonstrate the complex multifaceted characteristics of prolonged ED LOS. The potential effects are numerous and severe. Strategies to reduce this LOS may save costs associated with inpatient care, as well as ED costs, and they may prevent patient morbidity and mortality related to prolonged LOS [9]. Therefore, using Lean tools is recommended as it can identify targets for improving the efficiency of healthcare services. Lean tools seeks to reconfigure organizational processes to reduce waste and enhance productivity based on the application of specialized analytical techniques along with creating a culture of continuous improvement [25]. According to the NHS Institute for Innovation and Improvement (NHSIII) the application of Lean principles in healthcare, should remove duplicate processes and unnecessary procedures such as: recording patient details in multiple places, excessive waiting for staff, and uncoordinated, variable discharge processes resulting in a longer length of stay [26].

Organizational factors such as occupancy rate and handover management in shift exchange as well as miscommunication and lack of continuum-of-care, were found to be associated wtih ED LOS as can be concluded from the study results. Therefore, improvement interventions should take into account those types of factors in order to achieve valuable contributions to reducing this time. We believe our study contributes to a broader overview of this problem and to a better understanding of its causes.

Future study may help to assess whether a causal link exists between predictor variables and ED LOS using qualitative methods, and consequently, develop and evaluate data-based interventions to reduce ED LOS.
